# CellBRF: a feature selection method for single-cell clustering using cell balance and random forest

**DOI:** 10.1093/bioinformatics/btad216

**Published:** 2023-06-30

**Authors:** Yunpei Xu, Hong-Dong Li, Cui-Xiang Lin, Ruiqing Zheng, Yaohang Li, Jinhui Xu, Jianxin Wang

**Affiliations:** School of Computer Science and Engineering, Central South University, Changsha 410083, China; Hunan Provincial Key Lab on Bioinformatics, Central South University, Changsha 410083, China; School of Computer Science and Engineering, Central South University, Changsha 410083, China; Hunan Provincial Key Lab on Bioinformatics, Central South University, Changsha 410083, China; School of Computer Science and Engineering, Central South University, Changsha 410083, China; Hunan Provincial Key Lab on Bioinformatics, Central South University, Changsha 410083, China; School of Computer Science and Engineering, Central South University, Changsha 410083, China; Hunan Provincial Key Lab on Bioinformatics, Central South University, Changsha 410083, China; Department of Computer Science, Old Dominion University, Norfolk, VA 23529, United States; Department of Computer Science and Engineering, State University of New York at Buffalo, Buffalo, NY 14260, United States; School of Computer Science and Engineering, Central South University, Changsha 410083, China; Hunan Provincial Key Lab on Bioinformatics, Central South University, Changsha 410083, China

## Abstract

**Motivation:**

Single-cell RNA sequencing (scRNA-seq) offers a powerful tool to dissect the complexity of biological tissues through cell sub-population identification in combination with clustering approaches. Feature selection is a critical step for improving the accuracy and interpretability of single-cell clustering. Existing feature selection methods underutilize the discriminatory potential of genes across distinct cell types. We hypothesize that incorporating such information could further boost the performance of single cell clustering.

**Results:**

We develop CellBRF, a feature selection method that considers genes’ relevance to cell types for single-cell clustering. The key idea is to identify genes that are most important for discriminating cell types through random forests guided by predicted cell labels. Moreover, it proposes a class balancing strategy to mitigate the impact of unbalanced cell type distributions on feature importance evaluation. We benchmark CellBRF on 33 scRNA-seq datasets representing diverse biological scenarios and demonstrate that it substantially outperforms state-of-the-art feature selection methods in terms of clustering accuracy and cell neighborhood consistency. Furthermore, we demonstrate the outstanding performance of our selected features through three case studies on cell differentiation stage identification, non-malignant cell subtype identification, and rare cell identification. CellBRF provides a new and effective tool to boost single-cell clustering accuracy.

**Availability and implementation:**

All source codes of CellBRF are freely available at https://github.com/xuyp-csu/CellBRF.

## 1 Introduction

Recently developed single-cell RNA sequencing (scRNA-seq) technologies have emerged as a revolutionary tool for investigating transcriptomic cell-to-cell variation in gene expression profiling (Shalek et al. 2014). Single-cell clustering is critical for analyzing scRNA-seq data, as it is able to discover novel cell populations in the gene expression space, thereby further characterizing cell-to-cell heterogeneity. The single-cell clustering pipeline involves several steps that may impact the accuracy of clustering, including quality control, normalization, feature selection, dimensionality reduction, clustering analysis, and differential expression analysis (Luecken and Theis 2019). Among them, feature selection has been shown to be of crucial importance, as it can significantly improve clustering accuracy and enhance the biological significance of clustering results (Yang et al. 2021). Several studies aim to identify cell-type markers within single-cell clusters (Vargo and Gilbert 2020, Li et al. 2022). This differs from feature selection, which focuses on differentially expressed genes across all cells and thus accurately identifies all cell clusters. Cell-type marker identification focuses on finding genes that are specifically expressed in a particular cell cluster, defining a unique cell type.

A series of feature selection methods are developed for single-cell clustering. A commonly used approach is to consider genes with a higher-than-expected variance (also called highly variable genes, HVGs). Tools such as Seurat empirically fit the relationship between variance and mean expression for each gene to identify HVGs (Satija et al. 2015). Compared to previous studies that measure only individual genes based on their expression across cells, recent studies have focused more on the use of relationships among genes to classify cell types. geneBasis (Missarova et al. 2021) constructs a *k*-nearest neighbor (*k*-NN) graph as the reference and selects the genes with the maximum discrepancy at every iteration. DUBStepR (Ranjan et al. 2021) uses gene–gene correlation to identify an initial core set of genes and defines a graph-based measure of cell aggregation to optimize the number of genes. Highly Regional Genes (HRG) (Wu et al. 2022) chooses genes with expression levels regionally distributed on the cell–cell network or graph constructed based on cell similarity.

Unlike these methods, some recent studies use cluster labels of cells to guide the feature selection process, which leads to the idea of identifying cell type-related genes that enables better clustering performance. For example, Feats (Vans et al. 2021) first applies an agglomerative hierarchical clustering algorithm to obtain cluster labels, and then selects genes by calculating their ANOVA *F*-value. Similarly, FEAST (Su et al. 2021) relies on a combination of a consensus clustering algorithm and *F*-statistics for gene scoring. Despite the success of these methods, a common issue shared by them is that they do not consider the ability of features to distinguish cell types. This may lead to the selection of a subset of features that are not relevant to cell types.

To overcome these limitations, we present a feature selection method called CellBRF for single-cell clustering. The key idea of this method is to first predict cell labels by using a spectral clustering flow, followed by using a random forest algorithm with the predicted labels as input to estimate gene importance. Moreover, CellBRF proposes a class balancing strategy to mitigate the impact of skewed cell type distributions on feature importance measurement. We benchmark CellBRF with six recently proposed feature selection algorithms on 33 scRNA-seq datasets. We find that our proposed method outperforms other methods in both clustering accuracy and nearest-neighbors identification. Furthermore, we show that CellBRF can identify genes whose expression patterns can be used to characterize cell differentiation stages, cell subpopulations, and rare cell types.

## 2 Materials and methods

### 2.1 Algorithm of CellBRF

**Figure 1.**
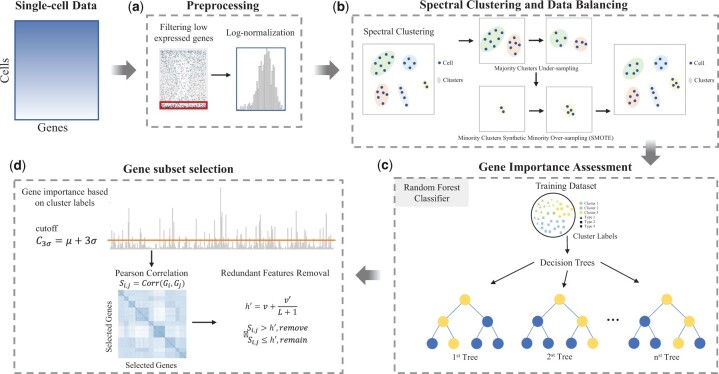
Overview of CellBRF workflow. (a) Preprocessing of scRNA-seq data includes lowly expressed genes removal and sequencing depth normalization followed by logarithm transformation. (b) A class balancing strategy that is capable of dealing with both over-sampling and under-sampling is proposed to balance data based on cluster labels obtained from the spectral clustering process. (c) Random forest model is used to measure gene importance, which is the average impurity reduction calculated from all decision trees in the forest. (d) An initial subset of genes is extracted based on the three-sigma rule and gene importance. Then, highly correlated genes are sequentially removed based on linear correlation analysis.

An overview of CellBRF is shown in Fig. 1. The main idea of CellBRF is to make use of the predicted labels in combination with random forests and a class balancing strategy to address the issue of skewed distribution of cell types in single-cell RNA-seq data. It consists of several steps, which are detailed in the following.

#### 2.1.1 Gene filtering and preprocessing

The scRNA-seq experiment produces an expression matrix **X** of size g×c, with *g* genes in rows, and *c* cells in columns. We first filter out genes that are expressed only in fewer than three cells, which provide little information for downstream analysis. Next, we perform the log-normalization procedure, including the calculation of cell-specific size factor based on the sequencing depths, normalization, and logarithm transformation on the expression matrix: $X^{\prime }={\;\text{log}\;}_{2}(X+1)$ (Fig. 1a).

#### 2.1.2 Spectral clustering and data balancing

With the preprocessed matrix $X^{\prime }$, CellBRF first predicts the cluster label of each cell by using a spectral clustering flow. Specifically, as done in the previous works (Wang et al. 2021), CellBRF performs the Principal Component Analysis (PCA) on $X^{{}^{\prime }}$ to obtain the top 50 principal components (PCs). Then, it constructs a cell graph, using Euclidean distance and *k*-NN algorithm with the nearest neighbor parameter *k* set to 15. Next, CellBRF uses the spectral clustering algorithm to cluster cells (in the cell graph) into *n* clusters. We choose this method because of its higher clustering performance and faster processing speed on large-scale data.

After that, CellBRF uniquely proposes a data balancing method that combines the cluster center-based under-sampling strategy and the Synthetic Minority Over-sampling Technique (SMOTE) (Chawla et al. 2002) to balance the data, which can effectively alleviate the issue of skewed distribution of cell types in scRNA-seq data (Fig. 1b). Specifically, CellBRF first defines a threshold $h=\frac{c}{n}$, where *c* is the total number of cells, *n* is the number of clusters. Then, it determines the cluster whose size is closest to this threshold as the central cluster ($C_{\text{central}}$) and applies the proposed strategies to balance different clusters. Compared to the central cluster, clusters containing fewer cells are identified as rare clusters, and clusters containing more cells are viewed as major clusters.

For rare clusters, SMOTE is used to perform oversampling to solve the imbalance problem. These synthetic cells are generated by randomly selecting one or more of the *k*-nearest neighbors for each cell in the rare clusters. Specifically, the following steps are used to synthesize artificial cells.

1. Let $C_{\text{rare}}$ be a rare cluster, for each cell $i\in C_{\text{rare}}$, the *k*-nearest neighbors are obtained by calculating the Euclidean distance between *i* and every other cell in cluster $C_{\text{rare}}$.
2. The sampling rate $R=\frac{T_{C_{\text{central}}}}{T_{C_{\text{rare}}}}$ is set individually according to the size of each rare cluster and determined central cluster, where $T_{C}$ is the size of cluster *C*. For each cell $i\in C_{\text{rare}}$, $R*T_{C_{\text{rare}}}$ cells (i.e. $i_{1},i_{2},\ldots ,i_{R}$) are randomly selected from their *k*-nearest neighbors, which is denoted as set ${\text{NN}}_{i}$.
3. For each cell $j\in {\text{NN}}_{i}(j=1,2,3,\ldots ,R)$, the following formula is used to generate a new cell: $i^{\prime }=i+\text{rand}(0,1)*|i-j|$, in which rand(0,1) represents the uniformly distributed random number between 0 and 1.

For major clusters in the data, CellBRF proposes a cluster center-based under-sampling strategy to balance the distribution of major clusters, which can reduce both the information loss and the variance of predicted labels. Specifically, CellBRF first calculates the center of each major cluster. Then, Euclidean distances between all cells in each major cluster and the cluster centers are calculated and used for cell sorting. Finally, CellBRF retains U=80% of the cells in each major cluster with the shortest distance from the cluster centers.

#### 2.1.3 Gene importance assessment

With the obtained cluster labels, the resulting balanced dataset is then fed into the random forest model (Breiman 2001) to compute the importance of each gene (Fig. 1c). Random Forest outputs feature importance which is computed based on the Gini impurity obtained from a set of decision trees. Specifically, CellBRF uses all cells and randomly selected genes to build each decision tree to ensure that the label accuracy of each decision tree is consistent. Decision trees are constructed by recursively evaluating different features and using at each node the feature that best splits the data with the lowest value of Gini impurity. For each decision tree, CellBRF calculates node importance using Gini impurity, assuming that each node has only two children (binary tree): where $I_{i}$ is the Gini impurity of node *i*, $p_{Ci}$ is the probability of cells belonging to cluster *C* at node *i*, *n* is the number of unique clusters, $N_{i}$ is the importance of node *i*, $w_{i}$ is the number of cells in node *i*, l(i) is the child node from left split on node *i*, and r(i) is the child node from right split on node *i*. The importance of feature *i* on a decision tree is then calculated as: where $M_{i}$ is the set of nodes split on feature *i*, *M* is the set of all nodes. The final gene importance is calculated as the average of the importance obtained from all trees.


(1) $$I_{i}=1-\sum_{C=1}^{n}p_{Ci}^{2}$$

(2) $$N_{i}=w_{i}I_{i}-w_{l(i)}I_{l(i)}-w_{r(i)}I_{r(i)}$$

(3) $$f_{i}=\frac{\sum_{a\in M_{i}}N_{a}}{\sum_{b\in M}N_{b}}$$

#### 2.1.4 Gene subset selection

CellBRF identifies a set of genes based on feature importance, and then removes highly linearly correlated redundant genes to optimize the gene set (Fig. 1d). Specifically, CellBRF selects genes whose importance is greater than or equal to μ+3σ, where μ and σ represent the mean and standard deviation of the importance of all genes, respectively. Since there are genes that are highly correlated and contain redundant information, CellBRF includes a step to automatically remove redundant genes. Specifically, we first generate an ordered gene list $G=\left\{G_{1},G_{2},\ldots ,G_{m}\right\}$ according to gene importance, where $G_{1}$ represents the gene with the highest importance, and *m* is the number of selected genes. CellBRF then sequentially calculates the linear correlation between genes $G_{i}$ and $G_{j}$ which is defined as $S_{i,j}=\text{Corr}(G_{i},G_{j})(i<j)$, where $\text{Corr}(G_{i},G_{j})$ is the Pearson correlation between $G_{i}$ and $G_{j}$. CellBRF removes highly redundant genes according to the threshold: where *v* is the initial correlation threshold (default 0.8), *L* is the length of the list of genes not yet analyzed, and $v^{\prime }$ is a parameter (default 0.1) that adjusts the threshold used for genes with high importance. If $S_{i,j}>h^{\prime }$ then gene $G_{j}$ will be removed, otherwise, both will be retained.


(4) $$h^{\prime }=v+\frac{v\prime }{L+1}$$

### 2.2 Single-cell RNA-seq datasets

We obtained 33 scRNA-seq datasets generated from different sequencing platforms, representing different biological scenarios (See details in Supplementary Table S1). These datasets are obtained from various public websites, including NCBI Gene Expression Omnibus (GEO), ArrayExpress, and Sequence Read Archive (SRA). The mouse bladder cells dataset, named Han, is obtained from the Mouse Cell Atlas project (Han et al. 2018). The 10X PBMC dataset is obtained from the website of 10X genomics (Zheng et al. 2017). The worm neuron cells dataset, named Cao, is sampled from the dataset generated by the sci-RNA-seq platform (single-cell combinatorial indexing RNA sequencing) (Cao et al. 2017).

### 2.3 Performance evaluation criteria

Two commonly used criteria, i.e. normalized mutual information (NMI) (Strehl and Ghosh 2002) and adjusted Rand index (ARI) (Rand 1971), are used to assess clustering performance. In addition, we use silhouette (Rousseeuw 1987) and *k*-nearest neighbor consistency to analyze the distribution of cells in the space composed of features selected by different methods. The value of the silhouette ranges from –1 to 1, where a high value means clusters are well apart from each other and distinguished.

For a given gene expression data containing *c* cells, *k*-nearest neighbors are computed for each cell based on Euclidean distance. Let F(i,j) be the identity of whether the cell types are consistent or not. If cell *i* and cell *j* belong to the same cell type, the corresponding F(i,j) is 1, and 0 otherwise. The *k*-nearest neighbor consistency is calculated as:


(5) $$F_{k}^{\prime }=\frac{\sum_{i=1}^{c}\sum_{j=1}^{k}F(i,j)}{ck}$$

The *k*-nearest neighbor consistency ranges from 0 to 1, where a high value indicates that the type of the cell is highly consistent with its *k*-nearest neighbors.

## 3 Results

### 3.1 Comparison of clustering performance with other feature selection strategies

We compare CellBRF with six state-of-the-art feature selection methods: a commonly used method [HVG (Satija et al. 2015)], two clustering-based feature selection methods [Feats (Vans et al. 2021), FEAST (Su et al. 2021)], a *k*-NN graph-based feature selection method geneBasisR (Missarova et al. 2021), a gene correlation-based feature selection method [DUBStepR (Ranjan et al. 2021)] and a cell–cell similarity-based method HRG (Wu et al. 2022). For Feast, geneBasis, and HVG, the number of selected genes is set to their default values of 2000, 50, and 2000, respectively. Feats iteratively increases the number of features and determines the optimal number of features based on the silhouette coefficient. Therefore, we set the number of features per increment to five on large datasets. For the other parameters of these methods, we used default values and benchmarked all genes of their respective selections.

**Figure 2.**
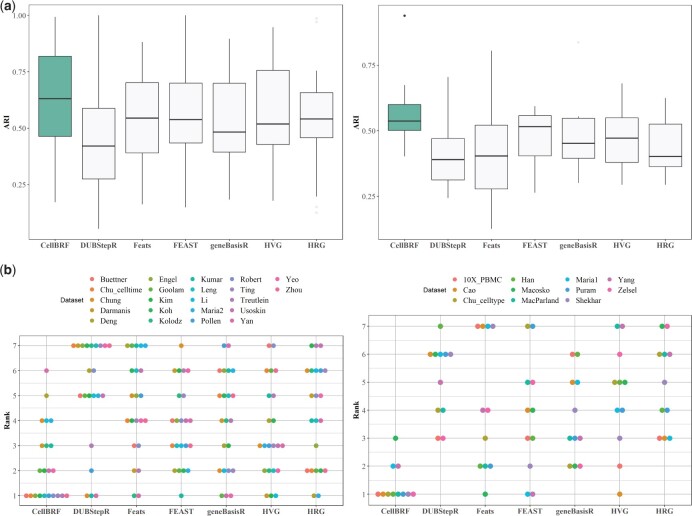
Benchmarking different feature selection methods. (a) Comparison of CellBRF with six state-of-the-art feature selection methods for single-cell clustering in terms of adjusted Rand index (ARI) on small datasets (left) and large datasets (right), respectively. (b) Rank distribution of feature selection methods. For each dataset, the methods are ranked from 1 to 7 by their ARI on small datasets (left) and large datasets (right), respectively.

To compare the performance of these methods, we evaluate the cell-type-discriminating performance of features via cell clustering. Specifically, since some methods select more genes, we first uniformly use PCA to reduce the data dimension, and then we apply a graph-based Louvain community detection algorithm in Seurat (Satija et al. 2015), which is a commonly used software toolkit for scRNA-seq clustering, to cluster cells. Larger datasets are more reflective of the true distribution of single cell types, i.e. are more imbalanced, than smaller datasets for specific research purposes. Because of that, we divide results into large and small datasets. The number of genes selected by each method is provided in Supplementary Table S2. The clustering results on all datasets are compared to the predefined annotation of cell types/classes and quantified using Normalized Mutual Information (NMI) and adjusted Rand index (ARI) (Supplementary Tables S3 and S4). In Fig. 2, we show the distribution of clustering accuracy and ranking on 21 small datasets and eleven large datasets, respectively. As shown in Fig. 2a, compared to other methods, CellBRF achieves significantly better average clustering performance, with an average ARI of 0.66 and 0.57 on small and large datasets, respectively, and with an average improvement of 0.12 compared to other methods, indicating that its selected genes have a better ability to cluster cells. Moreover, CellBRF is one of the top three algorithms on 17 of the 22 small datasets and all large datasets, respectively (Fig. 2b). Considering the possible impact of PCA, we use only those genes selected by different methods (without using PCA) to compare their clustering performance (Supplementary Tables S5 and S6). The experimental results suggest that CellBRF still achieves good performance. In summary, CellBRF substantially outperforms all other methods and is the highest-ranked algorithm on average across all datasets, especially large datasets.

### 3.2 CellBRF can accurately reconstruct cell neighborhoods

**Figure 3.**
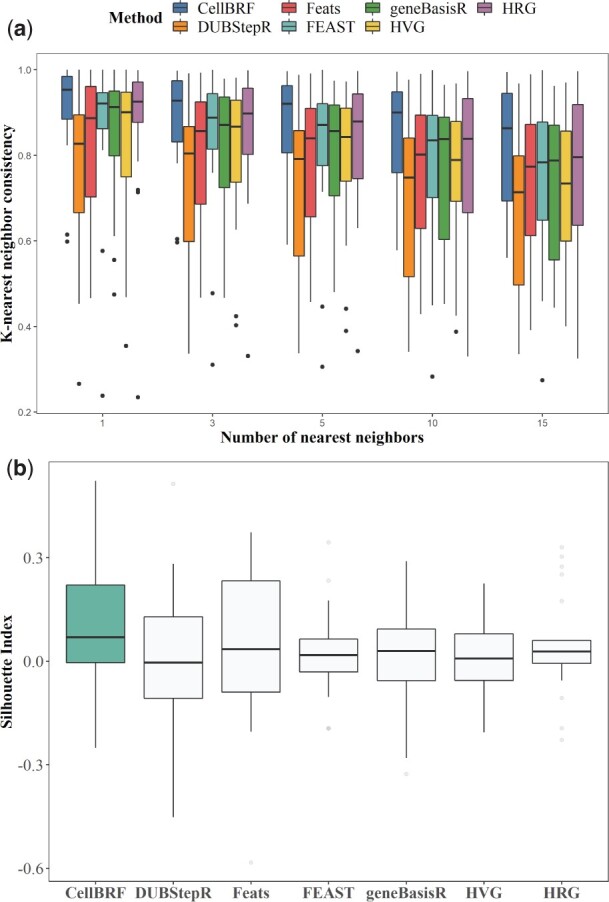
Analysis of cell types distribution in the feature spaces constructed based on different feature selection methods. All results are based on 22 small datasets with true labels. (a) Comparison of *k*-nearest neighbor consistency distributions in different feature spaces. (b) Comparison of silhouette index distributions in different feature spaces.

If features are well selected, it is expected that in the feature space, the neighbor of each cell can be accurately identified. We compare the performance of feature selection methods in identifying neighbors by using only the genes they have selected. Based on the known annotated cell type information, we use *k*-nearest neighbor consistency and silhouette coefficient to measure the spatial distribution of different cell types locally and globally in the feature space, respectively. Specifically, we first calculate the proportion of each cell’s *k*-nearest neighbors (k=1,3,5,10,15) that belong to the same type as an accuracy measure (Fig. 3a and Supplementary Table S7). Second, we use the average silhouette coefficient to measure the degree of separation between cells and cells from neighboring types (Fig. 3b and Supplementary Table S8). Since the silhouette coefficient needs to calculate the distance between cells, all experiments are performed on 22 small datasets for computational efficiency.

By comparing the *k*-nearest neighbor consistency, we find that CellBRF significantly outperforms all other methods with respect to different numbers of nearest neighbors. Moreover, CellBRF achieves better cell type separation on average than other methods in feature spaces. This indicates that CellBRF can effectively reduce the distance between cells of the same type, while separating cells from different types, thus leading to more accurate reconstruction of cell neighborhoods.

### 3.3 Data balancing improves clustering performance

**Figure 4.**
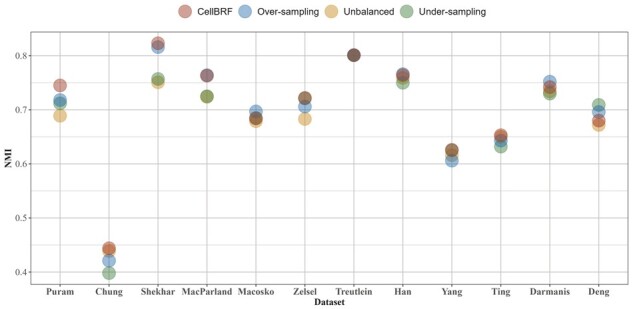
Clustering performance comparison of CellBRF in various aspects (under-sampling major clusters, over-sampling rare clusters, and without data balancing) in terms of NMI on top 12 imbalanced datasets.

To demonstrate the effectiveness of the class balancing strategy, we test the clustering performance of CellBRF in various scenarios (e.g. under-sampling major clusters, over-sampling rare clusters, and without data balancing). We define a Shannon entropy-based criterion, Balance Entropy (BE), to measure the degree of imbalance in different scRNA-seq datasets. For a given gene expression data containing *c* cells, *n* classes of size $T_{i}$, we can compute Shannon entropy as follows: $H=-\sum_{i=1}^{n}\frac{T_{i}}{c}\;\text{log}\;\frac{T_{i}}{c}$. This is equal to log n when all classes are balanced of the same size $\frac{c}{n}$. Therefore, we use the following measure of Balance Entropy (BE) for a dataset: $\text{BE}=1-\frac{H}{\;\text{log}\;n}$. The dataset’s imbalance degree increases as its BE value changes from 0 to 1. Then, we visualize clustering results on the top 12 imbalanced datasets (BE > 0.1) in terms of NMI (Fig. 4). The NMI and ARI values of each dataset are provided in Supplementary Table S9.

The experiments seem to suggest that over-sampling plays a more important role in top imbalanced datasets. However, the average clustering performance of under-sampling is higher overall. Combining both strategies has better clustering performance than using only one of them. Compared to not performing data balancing, CellBRF with data balancing achieves better performance on all 12 datasets in terms of NMI and better performance on 10 of the 12 datasets in terms of ARI. Moreover, data balancing significantly improves the clustering performance on Puram, Shekhar, MacParland, and Zelsel datasets, which have lower BE values, indicating that our balancing strategy is effective in handling imbalanced scRNA-seq data that can be used in random forests.

### 3.4 CellBRF identifies the better feature set based on the gene importance

**Figure 5.**
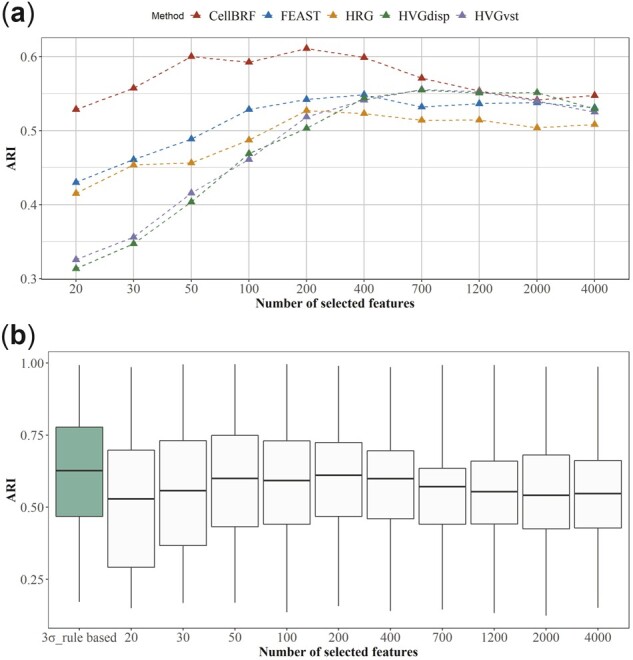
Clustering performance analysis of feature sets based on gene importance. (a) Average clustering performance comparison of ranking-based feature selection methods on 21 datasets with different feature set sizes (5–4000) in terms of ARI. (b) Clustering performance ranking distribution of feature sets with different feature set sizes (5–4000 and three-sigma rule) after ranking of gene importance based on CellBRF.

The key to ranking-based feature selection methods is whether the adopted gene importance measure can reflect its ability to distinguish cell types or not. Therefore, we compare CellBRF with four other ranking-based feature selection methods, including two variants of the HVG approach (HVGDisp, HVGvsp), HRG, and FEAST. We evaluate the clustering performance of each algorithm across a wide range of feature set sizes (20–4000) and then calculate the average ARI value across all datasets (Fig. 5a and Supplementary Table S10).

As shown in Fig. 5a, our results suggest that CellBRF achieves the best performance when the feature set size is 200, while the other methods need more features (200–2000) to attain their respective peak performance. At the same time, the genes ranked by CellBRF have remarkable clustering performance. This indicates that the importance calculated by CellBRF can effectively capture the roles of genes in cell clustering.

As shown above, choosing different sizes of the feature set can affect the clustering results. Ideally, we would like to select a small feature set that maximizes the separation between different cell types in the feature space. We consider using the three-sigma rule to identify features with better clustering performance without requiring the knowledge of cell-type labels. We also compare the clustering performance of the feature set determined by CellBRF and other feature sets with different sizes (Fig. 5b). The ARI values of each feature set are provided in Supplementary Table S11. The ARI distribution on different datasets indicates that CellBRF can identify the optimal feature set with the highest average clustering performance.

### 3.5 Case studies of CellBRF-selected genes in different biological scenarios

We analyze the biological and functional properties of selected genes with three case studies on cell differentiation stage identification, non-malignant cell subtype identification, and rare cell identification.

#### 3.5.1 CellBRF improves the identification of cell differentiation stages in a time-course dataset

**Figure 6.**
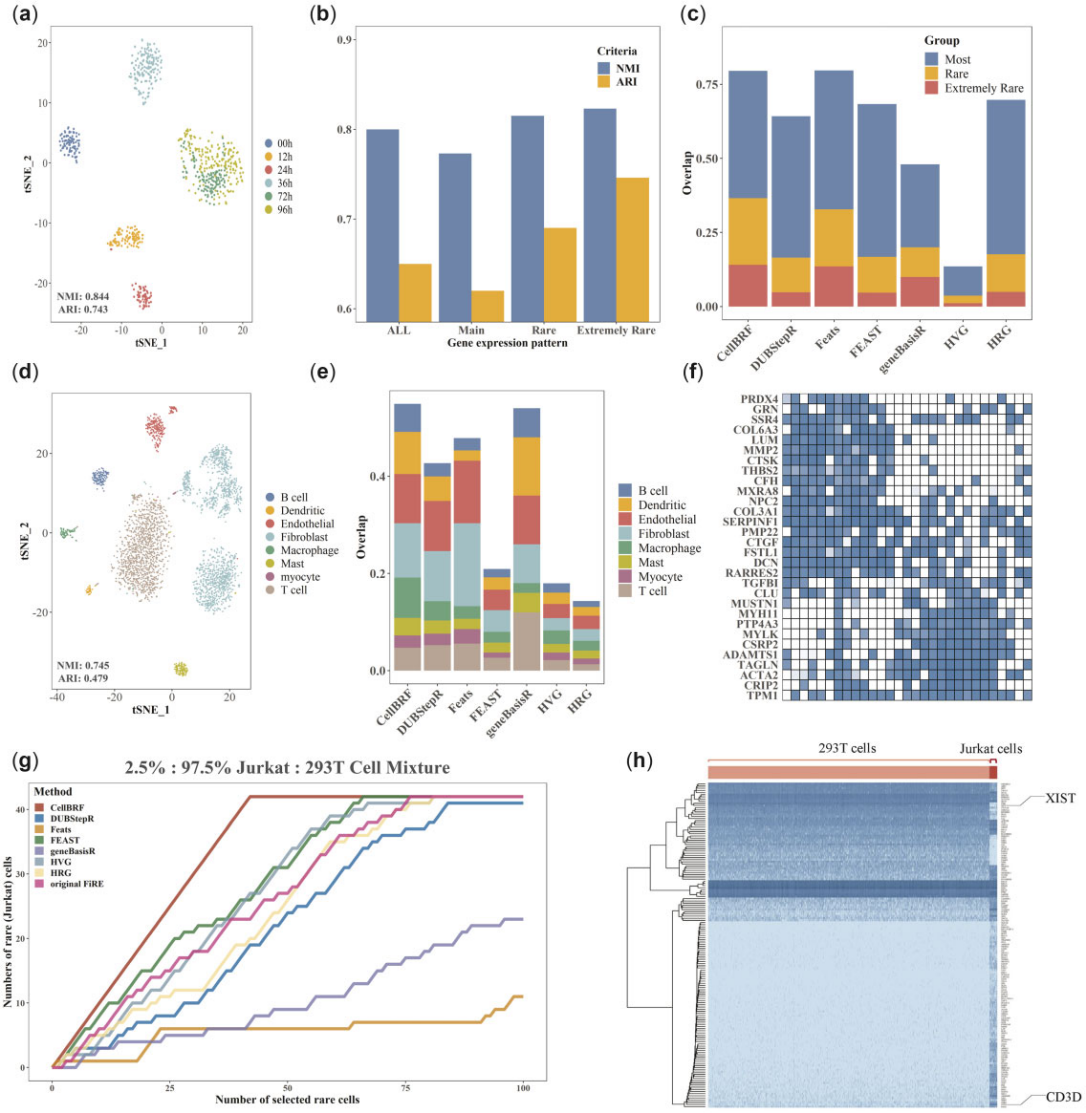
Case studies of CellBRF-selected genes in different biological scenarios. (a) t-SNE visualizations of time-course scRNA-seq data using features selected by CellBRF. (b) Comparison of clustering performance for genes with different expression patterns in terms of NMI and ARI. (c) Overlap of genes with different expression patterns in the results of each method. (d) t-SNE visualizations of the Puram dataset using features selected by CellBRF. (e) Overlap of marker genes for different cell types in the results of each method. (f) Heatmap of the top 30 genes that are most differentially expressed in fibroblasts from the CellBRF results. (g) Comparison of Jurkat cell number changes in cells sorted according to FiRE scores calculated based on different gene selection results on simulated dataset containing 2.5% Jurkat cells. (h) Expression heatmap of genes selected by CellBRF on simulated data with 2.5% Jurkat cells.

To demonstrate the ability of CellBRF to identify cell differentiation stages, we conduct further analysis on a real time-course dataset. Chu et al. (2016) perform scRNA-seq at six time points along the differentiation protocol to produce definitive endoderm (DE) cells from human embryonic stem (ES) cells (Chu dataset). We use t-SNE to visualize scRNA-seq data (Rtsne R package, version 0.15) with the genes selected by CellBRF (Fig. 6a). For t-SHE, default values of parameters are used. The visualization results for other methods are shown in Supplementary Fig. S1. Our experiments show that cells collected at 0 h, 12 h, and 24 h are individually tightly grouped in most visualizations except for geneBasisR and HVG. CellBRF is the only method that distinguishes cells well at stage 36 h from cells at other stages. To quantify results, we test both the separability of cell types (silhouette coefficient) and the accuracy of the *k*-NN classifier based on the t-SNE embedding results (Supplementary Table S12). For the *k*-NN classifier, we randomly select 80% of the cells for training and test the accuracy under different *k* values. Compared with other methods, CellBRF has the highest silhouette coefficient and accuracy, which indicates that the embedding results obtained by CellBRF can better distinguish different cell types.

Chu et al. (2016) identify genes with expression changes in this scRNA-seq time course data by using SCPattern (Leng et al. 2016), which calculates the posterior probability (PP) of each gene in different possible expression paths. A total of 3247 genes with different stage-specific expression patterns were identified and provided. To further analyze the expression characteristics of the genes selected by each method, we identify genes with specific expression patterns in the results. Specifically, we divide the expression patterns into three categories according to the number of genes: major expression patterns (greater than 100), rare expression patterns (less than 100 but greater than 10), and extremely rare expression patterns (less than 10). Compared to genes with major expression patterns, genes with rare expression patterns are characterized by bi/multi-modally distributed expression. We test the clustering performance of genes with different expression patterns (Fig. 6b). Our results indicate that these genes with rare expression patterns may have more discriminative information that can help identify the stages of cell differentiation. Then, we calculate the overlap percentage of different expression patterns with respect to the results for each method (Fig. 6c). Our study shows that genes with characteristic expression patterns dominate those from CellBRF, DUBStepR, Feats, FEAST, and HRG (higher than 60%). We further observe that the overlap percentage of genes with rare expression patterns in the CellBRF results is higher than those of other methods.

#### 3.5.2 CellBRF helps identify fibroblast subtypes in non-malignant cells from human tumor

To demonstrate the ability of CellBRF to cluster tumor cells, we further analyze the results of each method on the Puram dataset (Puram et al. 2017). This dataset consists of 3363 non-malignant cells grouped into eight major types: T cells, B/plasma cells, macrophages, dendritic cells, mast cells, endothelial cells, fibroblasts, and myocytes. The data with genes obtained by different feature selection methods are visualized using t-SNE (Fig. 6d and Supplementary Fig. S2). We perform a quantitative analysis for the visualization results, as we did in the previous section (Supplementary Table S13). CellBRF and HVG have approximate classifier accuracy. In addition, CellBRF also has the highest silhouette coefficient indicate that CellBRF can clearly distinguish different cell types. Furthermore, some of the rare cell types, such as dendritic cells and endothelial cells, also form tighter clusters and separate from other types.

For each cell type in the Puram dataset, we collect a total of 839 marker genes from the PanglaoDB (Franzén et al. 2019) database to analyze the composition of the selected gene features for each method (Fig. 6e). We find that the cell-type markers occupy the largest proportion in the results of CellBRF, and the cell-type marker distribution is relatively balanced, which reflects the advantage of CellBRF on imbalance dataset. On the contrary, although geneBasisR has a higher total proportion, it cannot identify the marker genes of Myocytes, and the proportions of different types of marker genes are quite different.

To test the ability to identify cell subtype marker genes, we analyze the expression of CellBRF-selected genes in fibroblasts. Specifically, we focus on the top 30 genes that are most differentially expressed in fibroblasts from the CellBRF results. For intuitive visualization, we classify fibroblasts according to Euclidean distance and equidistantly extract a smaller cell subset to visualize gene expression (Fig. 6f). To observe the states of fibroblasts in human tumors, we collect marker genes for two major subpopulations of fibroblasts, myofibroblasts and cancer-associated fibroblasts (CAFs). Interestingly, we find classical markers of myofibroblasts and CAFs including alpha smooth muscle actin (ACTA2) (Rockey et al. 2013), connective tissue growth factor (CTGF) (Xing et al. 2010), and matrix metalloproteinase 2 (MMP2) (Hassona et al. 2014) in the feature selection results of CellBRF, illustrating the ability of our method to identify cell subtypes.

#### 3.5.3 CellBRF enhances the ability to identify rare Jurkat cells in cell mixture experiments

Jindal et al. (2018) propose Finder of Rare Entities (FiRE) to assign a rareness score to each cell, thus allowing us to focus on rare cells. FiRE selects the 1000 most variable genes based on relative dispersion. We design a simulation experiment based on FiRE to evaluate the performance of selected genes in identifying rare cells. Specifically, we use a scRNA-seq data comprising 293T and Jurkat cells mixed in vitro in equal proportion (50%:50% Jurkat:293T Cell Mixture) (Zheng et al. 2017). Following the approach of Jindal et al. (2018), we simulate rare cells by reducing the proportion of Jurkat cells in the data. On the datasets with 1%, 1.5%, 2%, 2.5%, and 5% rare cells, we replace the feature selection step in FiRE with different feature selection methods and calculate the corresponding FiRE scores. The feature selection step in the FiRE algorithm is also included for comparison. After sorting cells according to different FiRE scores, we count and visualize the changes in the number of rare cells on simulated data with 2.5% Jurkat cells (Fig. 6g). The results on simulated datasets with 1%, 1.5%, 2%, and 5% rare cells are shown in Supplementary Fig. S3. CellBRF demonstrates better performance compared to other methods on each simulated dataset, i.e. almost all cells with high FiRE scores are rare Jurkat cells. At the same time, we find that the number of Jurkat cells identified by geneBasisR and Feats is small, which indicates that both are greatly affected by data imbalance.

We further observe the expression of CellBRF-selected genes on simulated data with 2.5% Jurkat cells. In Fig. 6h, we find that these genes are significantly differentially expressed in these two cell types, including the Jurkat cell type-specific marker CD3D and the 293T cell type-specific marker XIST. Overall, we show that CellBRF can effectively enhance the ability to identify rare cell types.

### 3.6 Parameter sensitivity analysis for CellBRF

To evaluate the stability of CellBRF in terms of parameters, we divide the main parameters into four groups in Supplementary Table S15 according to the steps in CellBRF and use different values to test each parameter while keeping all other parameters unchanged on five gold-standard scRNA-seq datasets (Supplementary Fig. S4). A more detailed summary and discussion of these parameters are included in Supplementary Section A. Our results suggest that the clustering performance of genes selected by CellBRF is often stable across different values of the parameters around our proposed default values.

### 3.7 Comparison of computational time

To assess the scalability of CellBRF, we collect and compare the runtime of each feature selection method on datasets with various sample sizes. Specifically, we use five datasets with various sizes (500–30 000 cells) as examples and record the runtime of different methods. All experiments are performed on a Linux machine with 40 CPU cores at 2.20 GHz, 256 GB of memory. For the results of the large datasets, there are significant variations in the runtime. Among them, our method outperforms most methods in terms of runtime across five datasets. Unlike the other methods, the runtime of DUBStepR, which is based on the gene network, is primarily dependent on the number of genes in the filtered data rather than the number of cells. On the other hand, HVG based on the mean and variance of gene expression is minimally affected by the number of cells. In contrast, geneBasisR and Feats are more computationally expensive on big data with a large number of cells due to the costly operations such as iterative selection of genes (geneBasisR) and iterative clustering to determine the optimal number of genes (Feats). In summary, CellBRF is a highly computationally efficient method for analyzing large-scale single-cell datasets.

## 4 Discussion

In this study, we develop a feature selection method CellBRF to boost single-cell clustering performance, which combines a novel class balancing strategy with a random forest model to evaluate gene importance guided by predicted labels. CellBRF optimizes the feature set based on gene importance and a redundant feature removal method. We show that CellBRF outperforms existing methods in terms of higher clustering accuracy. In addition, CellBRF is capable of selecting genes that can help identify cell subtypes, cell differentiation stages, and rare cell types.

Like previously developed methods, our approach also has limitations that need to be addressed in future research. One limitation is that the quality of the cluster labels can impact the performance of feature selection methods. If the cluster labels are of low quality, it is possible that the selected features will not accurately reflect the underlying biological patterns in the data. To address this issue, it may be useful to consider designing a better cell clustering algorithm that incorporates prior knowledge, such as pairwise constraints, to improve the accuracy. Another limitation is that, like other methods, CellBRF focuses on only a subset of the most important or informative genes and ignores the rest, which also has the risk of excluding informative genes that could be useful for the clustering analysis. It will be interesting to explore and consider the potential impact of these deleted genes on clustering performance in future studies.

Overall, CellBRF is a valuable tool that can both significantly improve the accuracy and interpretability of scRNA-seq clustering analyses and advance our understanding of the underlying biological patterns in the data. We expect that CellBRF can be used as a promising feature selection method for the accurate clustering of cells, and can be readily applied to other single-cell analysis pipelines.

## Supplementary Material

btad216_Supplementary_Data
