# Supplementary material for: CellBRF: a feature selection method for single-cell clustering using cell balance and random forest
Source: Bioinformatics. 2023 Jun 30;39(Suppl 1):i368–76. doi: 10.1093/bioinformatics/btad216 (PMC10311305; doi:10.1093/bioinformatics/btad216)
Supplement: btad216_Supplementary_Data [file btad216_supplementary_data.pdf]

Supplementary materials for *CellBRF: a feature selection method for single-cell clustering using cell balance and random forest*

List of supplementary materials

**Fig. S1**	The visual analysis of time-course scRNA-seq data.
**Fig. S2**	The visual analysis of Puram dataset.
**Fig. S3**	Analysis of simulated datasets consisting of Jurkat and 293T cells.
**Fig. S4**	Stability test of CellBRF under different parameter values.
**Fig. S5**	Stability test of CellBRF by using labels with different error rates.
**Table S1**	Detailed information of the datasets used in this study.
**Table S2**	Comparison of the number of genes selected by different methods on all data sets
**Table S3**	Comparison of CellBRF with six state-of-the-art gene selection methods for single cell clustering in terms of Normalized Mutual Information (NMI).
**Table S4**	Comparison of CellBRF with six state-of-the-art gene selection methods for single cell clustering in terms of adjusted Rand index (ARI).
**Table S5**	Comparison of CellBRF with six state-of-the-art gene selection methods for single cell clustering without PCA in terms of Normalized Mutual Information (NMI).
**Table S6**	Comparison of CellBRF with six state-of-the-art gene selection methods for single cell clustering without PCA in terms of adjusted Rand index (ARI).
**Table S7**	In the spaces constructed based on different feature selection methods, the distribution of k-nearest neighbor consistency with different number of neighbors on all twenty-two small datasets.
**Table S8**	Comparison of silhouettes in feature spaces constructed by different feature selection methods on twenty-two small datasets.
**Table S9**	Clustering performance comparison of CellBRF in various scenarios on all datasets in terms of NMI and ARI.
**Table S10**	Average clustering performance comparison of ranking-based feature selection methods on all datasets with different feature set sizes (20$\sim$4000) in terms of ARI.
**Table S11**	Clustering performance comparison of feature sets with different feature set sizes (20$\sim$4000 and three-sigma rule of thumb) on all datasets in terms of ARI.
**Table S12**	Comparison of t-SNE embedding results based on silhouette coefficient and classifier accuracy on the time-course dataset.
**Table S13**	Comparison of t-SNE embedding results based on silhouette coefficient and classifier accuracy on the human tumor dataset.
**Table S14**	The runtime (in seconds) of various feature selection methods on five datasets of different sizes.
**Table S15**	A summary list of parameters used in CellBRF.
**Section A**	Stability of CellBRF with respect to parameters.

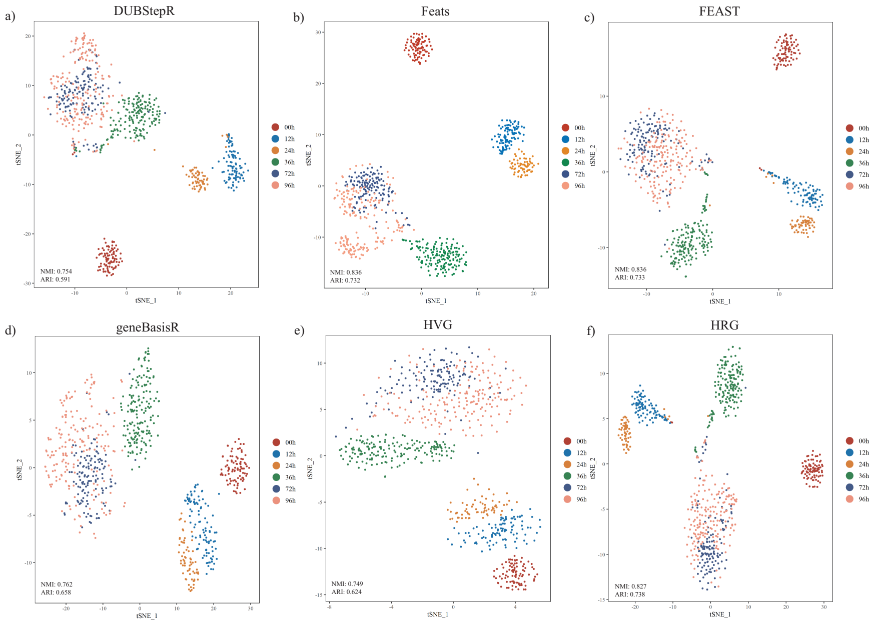

Fig. S1. The visual analysis of time-course scRNA-seq data. **(a-f)** t-SNE visualizations of time-course scRNA-seq data using features selected by six feature selection methods; **(a)** DUBStepR, **(b)** Feats, **(c)** FEAST, **(d)** geneBasisR, **(e)** HVG, and **(f)** HRG.

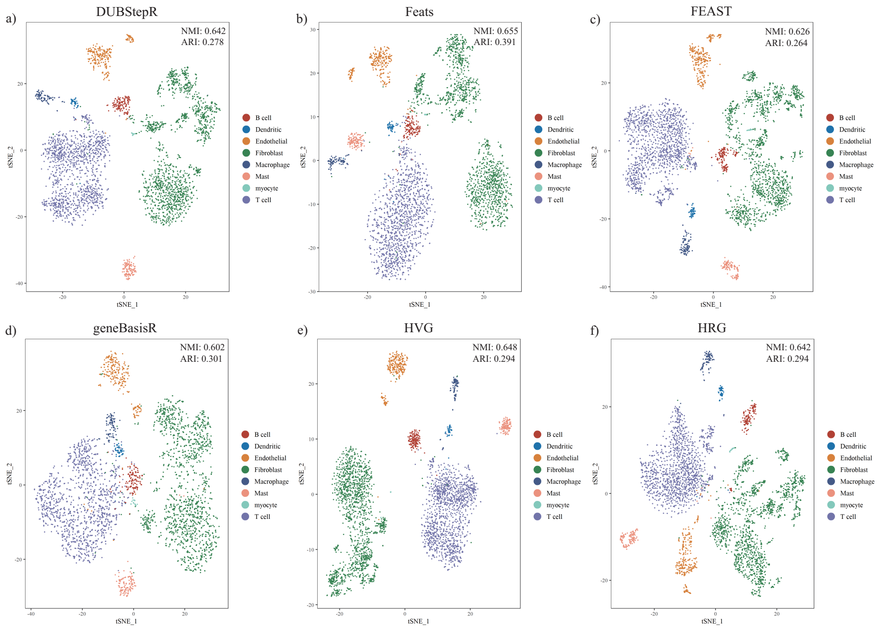

Fig. S2. The visual analysis of Puram dataset. **(a-f)** t-SNE visualizations of the Puram dataset using features selected by six feature selection methods; **(a)** DUBStepR, **(b)** Feats, **(c)** FEAST, **(d)** geneBasisR, **(e)** HVG, and **(f)** HRG.

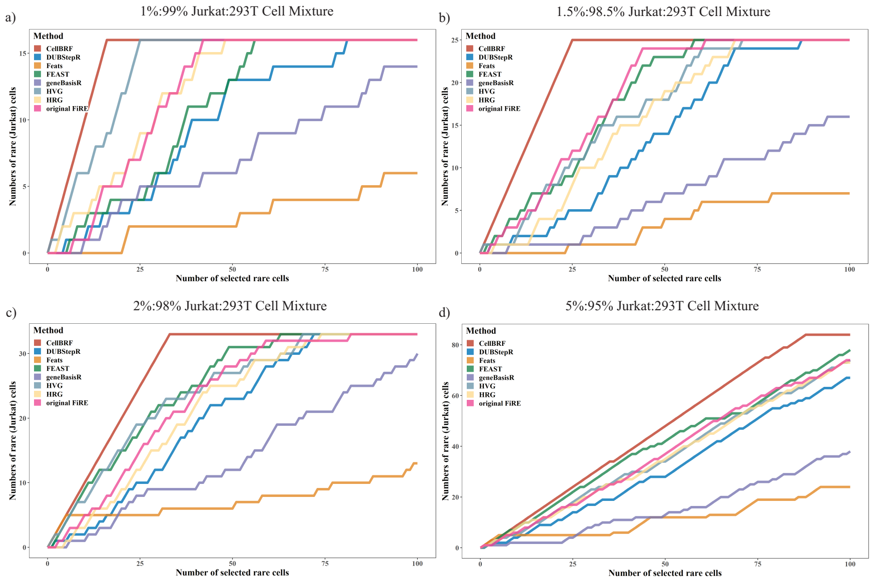

Fig. S3. Analysis of simulated datasets consisting of Jurkat and 293T cells. **(a-d)** Comparison of Jurkat cell number changes in cells sorted according to FiRE scores calculated based on different gene selection results on five simulated datasets containing different proportions of Jurkat cells; **(a)** 1% Jurkat cells, **(b)** 1.5% Jurkat cells, **(c)** 2% Jurkat cells, **(d)** 5% Jurkat cells.

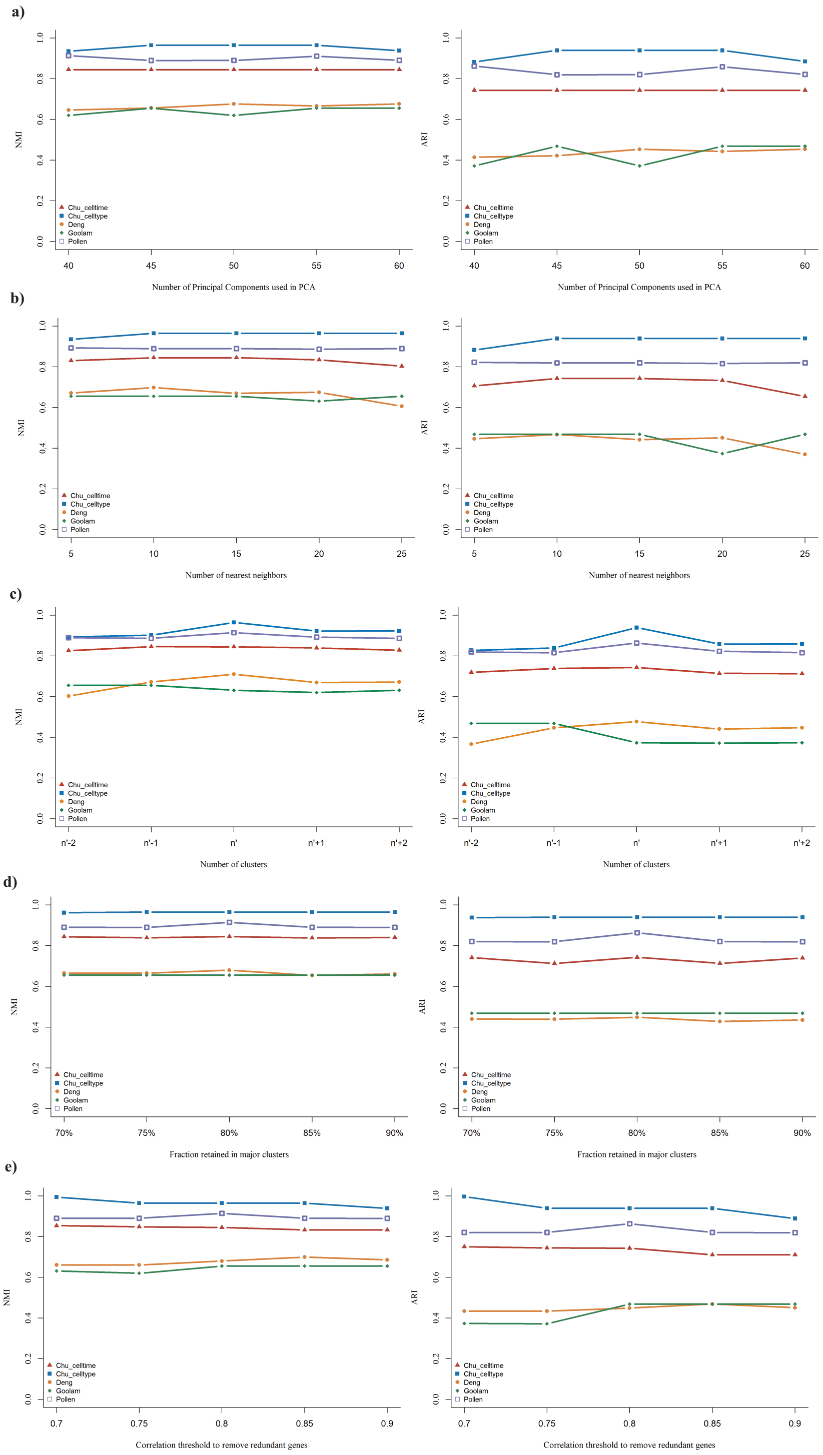

Fig. S4. The clustering performance of the final selected genes with different parameter values in terms of NMI and ARI. **a)** the number of principal components (PC) parameter (P), **b)** the neighbor parameter (k), **c)** the number of clusters (K), **d)** the fraction retained in major clusters (U), **e)** the initial correlation threshold (v).

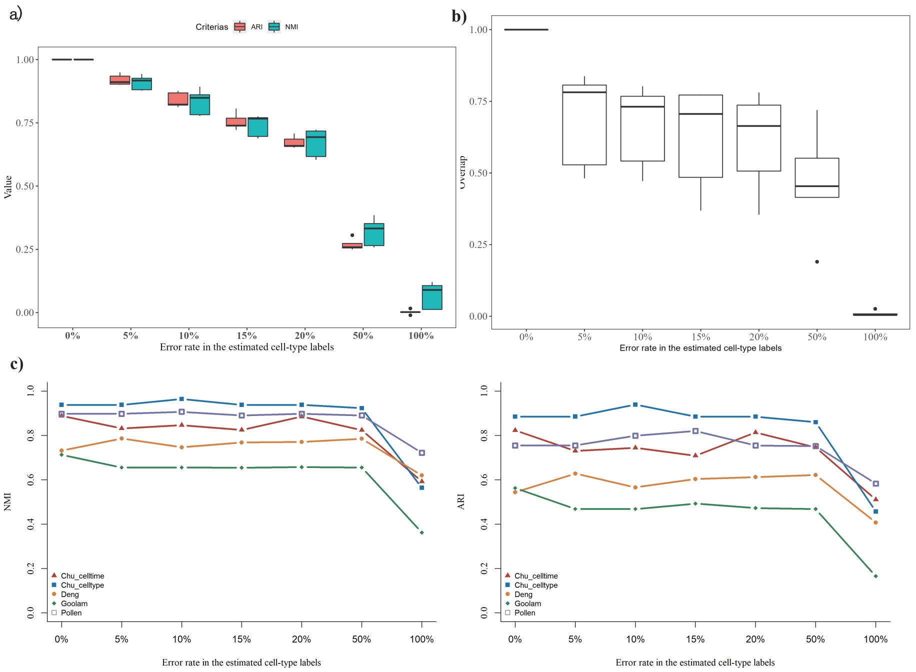

Fig. S5. Stability test of CellBRF by using labels with different error rates. **a)** The correspondence between various error rates and the evaluation criteria (NMI and ARI) on test datasets. **b)** Overlap comparison of genes selected based on labels with different error rates. **c)** The clustering performance of the final selected genes by using labels with various error rates in terms of NMI and ARI.

Table S1. Detailed information of the datasets used in this study.

	Dataset	Cells	Features	Types	Accession	Description
Small Datasets	Buettner	288	38293	3	E-MTAB-2805	mouse embryonic stem cells
	Chu1	758	19176	6	GSE75748	human pluripotent stem cells
	Chung	515	20345	5	GSE75688	human tumor and immune cells
	Darmanis	466	22085	9	GSE67835	human brain cells
	Deng	259	22958	10	GSE45719	mouse cells from different stages
	Engel	203	23337	4	GSE74596	mouse Natural killer T cells
	Goolam	124	41388	8	E-MTAB-3321	mouse cells from different stages
	Kim	89	32381	7	GSE55291	mouse induced pluripotent stem cells
	Koh	498	60483	9	GSM2257302	human embryonic stem cells
	Kolodz	704	38653	3	E-MTAB-2600	mouse embryonic stem cells
	Kumar	361	22394	4	GSE60749	mouse embryonic stem cells
	Leng	247	19084	3	GSE64016	human embryonic stem cells
	Li	561	57241	7	GSE81861	human cell lines
	Maria2	759	33694	7	GSE124731	human innate T cells
	Pollen	249	6982	11	SRP041736	human developing cortex cells
	Robert	194	23418	2	GSE74923	mouse leukemia cell line and primary CD8+ T-cells
	Ting	187	21583	7	GSE51372	mouse circulating tumor cells
	Treutlein	80	23271	5	GSE52583	mouse lung epithelial cells
	Usoskin	622	17772	4	GSE59739	mouse lumbar dorsal root ganglion
	Yan	90	20214	6	GSE36552	human embryonic stem cells
	Yeo	206	20345	3	GSE85908	human induced pluripotent stem cells
	Zhou	181	23937	8	GSE67120	mouse haematopoietic stem cells
Large Datasets	10X PBMC	4271	16653	8	10X genomics	peripheral blood mononuclear cells
	Cao	4186	13488	10	sci-RNA-seq platform	worm neuron cells
	Chu2	1018	19097	7	GSE75748	human pluripotent stem cells
	Han	2746	20670	16	Mouse Cell Atlas project	mouse bladder cells
	Macosko	14653	11422	39	GSE63473	mouse retina cells
	MacParland	8444	5000	11	GSE115469	human liver cells
	Maria1	1277	33694	7	GSE124731	human innate T cells
	Puram	3363	23686	8	GSE103322	non-malignant cells in Head and Neck Cancer
	Shekhar	27499	13166	19	GSE81905	mouse retina cells
	Yang	1119	46609	6	GSE90848	mouse bulge hair follicle stem cell, hair germ, basal transient amplifying cells (TACs) and dermal papilla
	Zelsel	3005	19486	9	GSE60361	mouse cerebral cortex cells

Table S2. Comparison of the number of genes selected by different methods on all data sets

	Dataset	CellBRF	DUBStepR	Feats	FEAST	geneBasisR	HVG	HRG
**Small Datasets**	Buettner	199	398	566	2000	50	2000	1435
	Chu1	249	394	192	2000	50	2000	2348
	Chung	246	241	535	2000	50	2000	831
	Darmanis	357	17	236	2000	50	2000	902
	Deng	281	904	464	2000	50	2000	804
	Engel	140	43	392	2000	50	2000	912
	Goolam	357	254	366	2000	50	2000	845
	Kim	250	249	294	2000	50	2000	1192
	Koh	321	892	38	2000	50	2000	2417
	Kolodz	259	291	40	2000	50	2000	1578
	Kumar	106	3587	415	2000	50	2000	2067
	Leng	133	1250	252	2000	50	2000	12073
	Li	242	3121	59	2000	50	2000	2337
	Maria2	122	566	96	2000	50	2000	1280
	Pollen	130	1718	11	2000	50	2000	298
	Robert	26	1074	498	2000	50	2000	952
	Ting	231	35	227	2000	50	2000	13214
	Treutlein	124	11	164	2000	50	2000	14722
	Usoskin	188	672	378	2000	50	2000	14145
	Yan	179	319	523	2000	50	2000	11551
	Yeo	176	92	148	2000	50	2000	831
	Zhou	249	395	519	2000	50	2000	960
**Large Datasets**	10X_PBMC	220	394	100	2000	50	2000	2527
	Cao	111	20	100	2000	50	2000	2744
	Chu2	227	1173	221	2000	50	2000	3118
	Han	249	144	500	2000	50	2000	3001
	Macosko	190	1674	400	2000	50	2000	6993
	MacParland	100	103	200	2000	50	2000	3367
	Maria1	127	13	5	2000	50	2000	1596
	Puram	277	553	234	2000	50	2000	5318
	Shekhar	155	104	100	2000	50	2000	4568
	Yang	262	379	102	2000	50	2000	1903
	Zelsel	318	247	242	2000	50	2000	7556

Table S3. Comparison of CellBRF with six state-of-the-art gene selection methods for single cell clustering in terms of Normalized Mutual Information (NMI).

	Dataset	CellBRF	DUBStepR	Feats	FEAST	geneBasisR	HVG	HRG
**Small Datasets**	Buettner	**0.583**	0.256	0.456	0.276	0.18	0.188	0.547
	Chu1	**0.844**	0.754	0.816	0.836	0.762	0.749	0.827
	Chung	0.444	0.41	0.44	0.431	**0.489**	0.475	0.447
	Darmanis	0.742	0.491	0.12	0.706	0.709	**0.747**	0.731
	Deng	0.68	0.597	0.607	**0.733**	0.704	0.697	0.719
	Engel	**0.808**	0.077	0.8	0.756	0.48	0.791	0.767
	Goolam	0.655	**0.682**	0.655	0.655	0.631	0.629	0.655
	Kim	0.569	0.49	0.436	0.316	0.525	**0.606**	0.295
	Koh	**0.991**	0.756	0.986	0.987	0.912	0.972	0.987
	Kolodz	0.667	0.499	0.61	0.653	0.583	**0.687**	0.641
	Kumar	0.989	**1**	0.835	**1**	0.902	0.931	0.963
	Leng	**0.946**	0.133	0.185	0.681	0.371	0.7	0.576
	Li	0.854	0.838	0.836	0.845	0.869	**0.918**	0.833
	Maria2	0.525	0.513	0.452	**0.611**	0.477	0.562	0.579
	Pollen	**0.914**	0.9	0.897	0.9	0.859	0.9	0.897
	Robert	**0.846**	0.599	0.634	0.615	0.764	0.606	0.591
	Ting	**0.653**	0.263	0.613	0.586	0.602	0.623	0.588
	Treutlein	**0.801**	0.509	0.52	0.366	0.397	0.272	0.3
	Usoskin	**0.786**	0.684	0.683	0.703	0.676	0.711	0.642
	Yan	0.691	**0.797**	0.721	0.721	0.661	0.767	0.721
	Yeo	0.815	0.558	0.691	0.681	**0.829**	0.801	0.703
	Zhou	0.717	0.444	0.724	0.712	0.542	0.596	**0.73**
	Mean	**0.751**	0.557	0.624	0.671	0.633	0.679	0.67
**Large Datasets**	10X_PBMC	0.736	0.734	0.558	0.735	0.668	0.719	**0.739**
	Cao	0.607	0.397	0.232	0.628	0.562	0.632	**0.656**
	Chu2	**0.964**	0.812	0.893	0.788	0.88	0.87	0.787
	Han	0.764	0.652	0.751	0.774	0.711	**0.775**	0.772
	Macosko	0.684	0.642	0.68	0.691	0.678	**0.727**	0.648
	MacParland	**0.763**	0.663	0.678	0.722	0.668	0.694	0.708
	Maria1	0.626	0.34	0.548	**0.634**	0.553	0.602	0.58
	Puram	**0.745**	0.642	0.64	0.626	0.602	0.648	0.642
	Shekhar	**0.823**	0.646	0.39	0.813	0.638	0.807	0.776
	Yang	0.626	0.538	0.609	**0.644**	0.565	0.542	0.577
	Zelsel	**0.722**	0.668	0.675	0.647	0.668	0.656	0.677
	Mean	**0.733**	0.612	0.605	0.7	0.654	0.698	0.687

Table S4. Comparison of CellBRF with six state-of-the-art gene selection methods for single cell clustering in terms of adjusted Rand index (ARI).

	Dataset	CellBRF	DUBStepR	Feats	FEAST	geneBasisR	HVG	HRG
**Small Datasets**	Buettner	**0.615**	0.199	0.429	0.284	0.183	0.178	0.544
	Chu1	**0.743**	0.591	0.703	**0.733**	0.658	0.624	0.738
	Chung	0.172	**0.269**	0.163	0.149	0.232	0.198	0.151
	Darmanis	0.625	0.387	0.042	0.537	0.619	**0.751**	0.587
	Deng	0.449	0.358	0.417	0.524	0.492	0.464	**0.538**
	Engel	**0.778**	0.054	0.72	0.681	0.36	0.758	0.693
	Goolam	0.468	0.455	0.468	0.468	**0.473**	0.45	0.468
	Kim	0.357	0.298	0.25	0.152	0.32	**0.373**	0.125
	Koh	**0.991**	0.642	0.986	0.987	0.892	0.947	0.987
	Kolodz	0.443	0.292	0.415	0.423	0.442	**0.493**	0.419
	Kumar	0.993	**1**	0.797	**1**	0.896	0.935	0.971
	Leng	**0.966**	0.101	0.165	0.706	0.419	0.727	0.475
	Li	0.703	0.7	0.698	0.706	0.864	**0.898**	0.667
	Maria2	0.438	0.349	0.345	**0.473**	0.348	0.45	**0.499**
	Pollen	**0.863**	0.832	0.755	0.83	0.706	0.83	0.755
	Robert	**0.899**	0.5	0.585	0.567	0.835	0.46	0.519
	Ting	**0.462**	0.14	0.433	0.405	0.422	0.42	0.374
	Treutlein	**0.816**	0.526	0.53	0.234	0.444	0.33	0.197
	Usoskin	0.637	0.503	0.477	0.54	**0.678**	0.544	0.454
	Yan	0.609	**0.691**	0.628	0.628	0.593	0.673	0.628
	Yeo	0.82	0.58	0.64	0.611	**0.832**	0.82	0.612
	Zhou	**0.595**	0.243	0.583	0.523	0.386	0.379	0.582
	Mean	**0.656**	0.442	0.51	0.553	0.55	0.577	0.545
**Large Datasets**	10X_PBMC	**0.606**	0.58	0.427	0.58	0.538	0.582	0.58
	Cao	**0.402**	0.243	0.19	0.361	0.346	**0.402**	0.381
	Chu2	**0.939**	0.705	0.828	0.594	0.838	0.681	0.625
	Han	**0.594**	0.477	0.587	0.581	0.552	0.571	0.574
	Macosko	0.537	0.405	**0.676**	0.536	0.555	0.529	0.367
	MacParland	**0.524**	0.39	0.404	0.378	0.4	0.356	0.36
	Maria1	**0.533**	0.253	0.435	0.534	0.448	0.472	0.477
	Puram	**0.479**	0.278	0.328	0.264	0.301	0.294	0.294
	Shekhar	**0.675**	0.377	0.126	0.516	0.452	0.514	0.423
	Yang	0.411	0.347	0.401	**0.431**	0.39	0.311	0.341
	Zelsel	**0.544**	0.465	0.462	0.445	0.543	0.442	0.402
	Mean	**0.568**	0.411	0.442	0.475	0.488	0.468	0.439

Table S5. Comparison of CellBRF with six state-of-the-art gene selection methods for single cell clustering without PCA in terms of Normalized Mutual Information (NMI).

	Dataset	CellBRF	DUBStepR	Feats	FEAST	geneBasisR	HVG	HRG
**Small Datasets**	Buettner	0.2157	0.0671	0.0511	0.0511	0.2317	0.0000	**0.2357**
	Chu1	**0.8824**	0.7592	0.7394	0.7889	0.7695	0.7110	0.7603
	Chung	0.3843	0.3741	0.4352	0.3910	0.4124	0.3885	**0.4531**
	Darmanis	0.7994	0.4850	0.7870	0.7780	0.7065	0.7117	**0.8157**
	Deng	**0.6726**	0.6230	0.4846	0.5968	0.6656	0.5999	0.6439
	Engel	**0.8100**	0.1262	0.4218	0.7040	0.7530	0.4926	0.7364
	Goolam	0.6552	0.6552	**0.6824**	0.6552	0.4921	0.6339	0.6552
	Kim	**0.5636**	0.4653	0.4661	0.3963	0.4778	0.5153	0.3306
	Koh	**0.9476**	0.7263	0.8878	0.8808	0.8690	0.8024	0.8675
	Kolodz	0.7156	0.5497	0.7272	**0.7568**	0.6532	0.6559	0.6646
	Kumar	**0.9451**	0.8431	0.8548	0.8432	0.8885	0.8422	0.9201
	Leng	**0.9030**	0.1020	0.0000	0.6876	0.4629	0.0000	0.0000
	Li	**0.8662**	0.8407	0.6223	0.8128	0.8046	0.8326	0.8377
	Maria2	**0.5146**	0.2876	0.4363	0.2426	0.4951	0.3365	0.2682
	Pollen	0.8896	0.7246	0.8886	0.7787	0.8274	0.7150	**0.9065**
	Robert	0.8695	0.8744	0.8744	0.6914	0.5012	**0.9269**	0.8247
	Ting	0.5955	0.2551	**0.6254**	0.6136	0.5994	0.3219	0.4670
	Treutlein	0.7570	0.7180	**0.7811**	0.5748	0.3497	0.5275	0.3043
	Usoskin	0.7793	0.7194	0.4262	0.8092	0.7617	**0.8399**	0.5034
	Yan	0.6721	**0.7972**	**0.7972**	0.7211	0.7211	**0.7972**	0.7211
	Yeo	**0.8238**	0.6809	**0.8238**	0.7939	0.7201	0.5060	0.8011
	Zhou	0.6285	0.4919	0.5919	0.6379	0.2734	0.5117	**0.6518**
	Mean	**0.7223**	0.5530	0.6093	0.6457	0.6107	0.5759	0.6077
**Large Datasets**	10X_PBMC	0.7630	**0.7872**	0.7264	0.7438	0.6694	0.7579	0.7107
	Cao	**0.5543**	0.3775	0.1826	0.3329	0.4982	0.3520	0.3165
	Chu2	**0.9907**	0.8887	0.9156	0.8919	0.9570	0.8856	0.8958
	Han	**0.7504**	0.6189	0.6955	0.6896	0.7099	0.7149	0.6840
	Macosko	**0.7139**	0.5561	0.6807	0.6266	0.7034	0.6776	0.4718
	MacParland	**0.7472**	0.6549	0.7241	0.6933	0.6874	0.7041	0.5774
	Maria1	**0.6199**	0.3663	0.2294	0.2375	0.4956	0.4368	0.2385
	Puram	0.7918	0.7169	0.7220	0.8261	0.6654	0.7463	**0.8294**
	Shekhar	0.8073	0.7147	0.4766	0.7971	0.7378	**0.8091**	0.5112
	Yang	**0.6604**	0.6324	0.5947	0.6460	0.5612	0.5607	0.6451
	Zelsel	0.7211	0.6949	0.6748	0.6303	0.7241	**0.7308**	0.6448
	Mean	**0.7382**	0.6371	0.6020	0.6468	0.6736	0.6705	0.5932

Table S6. Comparison of CellBRF with six state-of-the-art gene selection methods for single cell clustering without PCA in terms of adjusted Rand index (ARI).

	Dataset	CellBRF	DUBStepR	Feats	FEAST	geneBasisR	HVG	HRG
**Small Datasets**	Buettner	0.1769	0.0644	0.0028	0.0028	**0.2490**	0.0000	0.2166
	Chu1	**0.7363**	0.6353	0.6053	0.6637	0.6631	0.5578	0.6322
	Chung	0.0957	0.1859	0.1821	0.1393	**0.2140**	0.1458	0.1721
	Darmanis	0.7775	0.3954	**0.7838**	0.7466	0.6226	0.6595	0.7809
	Deng	**0.4660**	0.3942	0.2707	0.3899	0.4653	0.3930	0.4167
	Engel	**0.7691**	0.0972	0.2698	0.6627	0.7076	0.2940	0.6941
	Goolam	**0.4682**	**0.4682**	0.4551	**0.4682**	0.2931	0.4437	**0.4682**
	Kim	**0.3341**	0.3003	0.2566	0.1680	0.2703	0.3100	0.1278
	Koh	**0.9197**	0.5469	0.8228	0.7786	0.8074	0.6316	0.7495
	Kolodz	0.5304	0.3910	**0.5975**	0.5839	0.5040	0.4940	0.4393
	Kumar	**0.9562**	0.7024	0.7060	0.7027	0.8892	0.6999	0.9283
	Leng	**0.9124**	0.1078	0.0000	0.5894	0.4458	0.0000	0.0000
	Li	0.7562	0.7334	0.4845	0.7170	0.7073	**0.7774**	0.7337
	Maria2	**0.3728**	0.1798	0.3078	0.1547	0.3525	0.2218	0.1686
	Pollen	0.7489	0.5044	0.7791	0.5823	0.6853	0.4678	**0.7988**
	Robert	0.9172	0.9188	0.9188	0.6942	0.4117	**0.9590**	0.8795
	Ting	0.4286	0.0939	0.3879	0.3529	**0.4367**	0.1703	0.2674
	Treutlein	0.7834	0.7072	**0.7918**	0.5550	0.3881	0.5667	0.3521
	Usoskin	0.7109	0.6646	0.3363	0.8110	0.8249	**0.8361**	0.3511
	Yan	0.5926	**0.6911**	**0.6911**	0.6276	0.6276	**0.6911**	0.6276
	Yeo	**0.8323**	0.6466	**0.8323**	0.8194	0.6817	0.3684	0.8195
	Zhou	0.4237	0.2426	0.3779	0.3844	0.1161	0.2840	**0.4541**
	Mean	**0.6231**	0.4396	0.4936	0.5270	0.5165	0.4533	0.5035
**Large Datasets**	10X_PBMC	0.7530	**0.7558**	0.7148	0.6234	0.5978	0.6996	0.5493
	Cao	**0.3340**	0.2394	0.1375	0.1998	0.2981	0.1546	0.1765
	Chu2	**0.9935**	0.8004	0.7755	0.8027	0.9644	0.7349	0.8022
	Han	**0.6588**	0.4608	0.5067	0.4783	0.5462	0.5733	0.4610
	Macosko	0.5451	0.4499	0.5297	0.3865	**0.7264**	0.4951	0.3693
	MacParland	**0.5056**	0.3473	0.4864	0.4816	0.4974	0.4535	0.3554
	Maria1	**0.4978**	0.2222	0.1631	0.1128	0.3758	0.2859	0.1127
	Puram	0.6564	0.4737	0.6256	**0.7382**	0.4101	0.5094	0.7340
	Shekhar	0.6390	0.4654	0.1701	0.6442	0.6639	**0.6644**	0.3883
	Yang	**0.5430**	0.5039	0.4641	0.4278	0.3944	0.3668	0.4216
	Zelsel	0.6170	0.6940	0.6918	0.6025	0.6554	**0.7415**	0.5939
	Mean	**0.6130**	0.4921	0.4787	0.4998	0.5573	0.5163	0.4513

Table S7. In the spaces constructed based on different feature selection methods, the distribution of k-nearest neighbor consistency with different number of neighbors on all twenty-two small datasets.

k	CellBRF	DUBStepR	Feats	FEAST	geneBasisR	HVG	HRG
1	**0.9112± 0.1109**	0.7687± 0.1915	0.8445± 0.2155	0.8709± 0.1685	0.8438± 0.1579	0.8290± 0.1808	0.8755± 0.1664
3	**0.8916± 0.1159**	0.7455± 0.1889	0.8359± 0.1877	0.8445± 0.1637	0.8160± 0.1618	0.8105± 0.1665	0.8561± 0.1516
5	**0.8770± 0.1222**	0.7298± 0.1917	0.8226± 0.1849	0.8261± 0.1679	0.7981± 0.1636	0.7930± 0.1630	0.8343± 0.1555
10	**0.8508± 0.1342**	0.6942± 0.1948	0.7959± 0.1849	0.7871± 0.1767	0.7628± 0.1733	0.7538± 0.1651	0.7951± 0.1718
15	**0.8252± 0.1473**	0.6671± 0.1956	0.7662± 0.1933	0.7544± 0.1871	0.7312± 0.1788	0.7205± 0.1673	0.7649± 0.1868

Table S8. Comparison of silhouettes in feature spaces constructed by different feature selection methods on twenty-two small datasets.

Dateset	CellBRF	DUBStepR	Feats	FEAST	geneBasisR	HVG	HRG
Buettner	-0.0295	-0.0257	0.0118	-0.0058	-0.0346	-0.0011	**0.0217**
Chu1	**0.0733**	0.0226	-0.0993	-0.001	-0.1229	-0.0134	0.0012
Chung	-0.1837	-0.0984	-0.12	**-0.0316**	-0.2258	-0.171	-0.04
Darmanis	**0.0651**	-0.0648	-0.5828	0.0466	0.0339	0.0471	0.0619
Deng	**0.0962**	0.0297	0.0459	0.0552	0.0265	0.0657	0.0551
Engel	**0.0567**	-0.2176	0.0342	-0.0079	0.0377	-0.0201	0.0027
Goolam	-0.2511	-0.1361	-0.2039	-0.1949	**-0.0596**	-0.0825	-0.2276
Kim	**-0.0097**	-0.1099	-0.19	-0.1938	-0.2803	-0.1399	-0.1945
Koh	0.0537	**0.1187**	0.0353	0.043	0.0982	0.0167	0.0424
Kolodz	0.0132	-0.0043	**0.1224**	0.0581	-0.0477	-0.0092	-0.0019
Kumar	**0.328**	0.1156	0.277	0.1558	0.1557	0.0966	0.1743
Leng	**0.1517**	-0.1492	-0.0302	-0.0007	0.0784	-0.0032	-0.0071
Li	**0.1184**	-0.0028	0.0932	-0.0275	0.0378	0.0838	0.0307
Maria2	-0.0581	-0.0575	-0.059	-0.055	**-0.0267**	-0.0677	-0.0555
Pollen	**0.3501**	0.1623	0.3123	0.1759	0.2213	0.1312	0.303
Robert	**0.5226**	0.2822	0.278	0.037	0.0098	0.0376	0.33
Ting	**-0.0874**	-0.4521	-0.1362	-0.1037	-0.1945	-0.201	-0.1061
Treutlein	0.1912	**0.5137**	0.2315	0.0668	0.0345	0.0431	0.0562
Usoskin	0.2307	0.1554	**0.2332**	0.0832	0.1015	0.0933	0.0255
Yan	**0.3752**	0.2402	0.3734	0.3442	0.2897	0.2256	0.2742
Yeo	**0.3263**	0.1316	0.2863	0.2334	0.2102	0.1851	0.2517
Zhou	0.0362	-0.2025	0.027	-0.0628	-0.3269	-0.2061	**0.0439**
Mean	**0.1077**	0.0114	0.0427	0.0279	0.0007	0.005	0.0474

Table S9. Clustering performance comparison of CellBRF in various scenarios on all datasets in terms of NMI and ARI.

	Unbalanced		Under-sampling		Over-sampling		CellBRF		Balance Entropy (BE)
Dataset	NMI	ARI	NMI	ARI	NMI	ARI	NMI	ARI	–
Puram	0.689	0.410	0.712	0.460	0.718	0.416	**0.745**	**0.479**	0.339
Chung	0.439	0.162	0.398	0.110	0.421	**0.172**	**0.444**	**0.172**	0.279
Shekhar	0.751	0.476	0.757	0.484	0.816	0.673	**0.823**	**0.675**	0.245
MacParland	0.724	0.449	0.725	0.427	**0.764**	**0.534**	0.763	0.524	0.236
Macosko	0.679	0.550	0.685	**0.572**	**0.697**	0.558	0.684	0.537	0.196
Zelsel	0.683	0.448	0.721	**0.565**	0.706	0.528	**0.722**	0.544	0.188
Treutlein	**0.801**	**0.816**	**0.801**	**0.816**	**0.801**	**0.816**	**0.801**	**0.816**	0.181
Han	0.759	0.614	0.750	0.608	**0.766**	**0.616**	0.764	0.594	0.173
Yang	0.616	0.387	0.625	**0.442**	0.606	0.381	**0.626**	0.411	0.147
Ting	0.650	0.447	0.632	0.445	0.643	0.453	**0.653**	**0.462**	0.125
Darmanis	0.734	0.630	0.730	0.607	**0.752**	**0.632**	0.742	0.625	0.121
Deng	0.672	0.447	**0.709**	**0.480**	0.696	0.472	0.680	0.449	0.113
Goolam	**0.655**	**0.468**	**0.655**	**0.468**	**0.655**	**0.468**	**0.655**	**0.468**	0.096
Kim	**0.569**	**0.357**	**0.569**	**0.357**	**0.569**	**0.357**	**0.569**	**0.357**	0.089
Cao	0.605	0.372	**0.637**	**0.465**	0.582	0.364	0.607	0.402	0.086
Yan	**0.691**	**0.609**	**0.691**	**0.609**	**0.691**	**0.609**	**0.691**	**0.609**	0.086
10X PBMC	**0.758**	**0.747**	0.716	0.548	0.719	0.559	0.736	0.606	0.084
Li	**0.856**	**0.709**	0.825	0.680	0.853	**0.709**	0.854	0.703	0.082
Usoskin	0.761	0.604	0.773	**0.644**	0.760	0.611	**0.786**	0.637	0.046
Pollen	0.890	0.820	0.897	0.755	0.886	0.816	**0.914**	**0.863**	0.038
Chu_celltime	**0.848**	0.721	0.844	0.741	0.843	0.722	0.844	**0.743**	0.034
Kolodz	**0.669**	0.441	0.666	0.441	0.661	0.439	0.667	**0.443**	0.028
Chu_celltype	0.938	0.885	0.935	0.882	**0.964**	**0.940**	**0.964**	0.939	0.025
Koh	**0.995**	**0.995**	**0.995**	**0.995**	**0.995**	**0.995**	0.991	0.991	0.022
Maria1	0.579	0.472	**0.628**	**0.535**	0.614	0.526	0.626	0.533	0.016
Maria2	0.472	0.373	**0.528**	**0.445**	0.477	0.375	0.525	0.438	0.014
Engel	**0.813**	**0.779**	0.804	0.767	0.811	0.734	0.808	0.778	0.013
Zhou	**0.740**	**0.598**	0.710	0.578	0.602	0.428	0.717	0.595	0.010
Robert	0.598	0.585	**0.846**	**0.899**	0.596	0.575	**0.846**	**0.899**	0.006
Leng	0.848	0.870	**0.946**	**0.966**	0.931	0.955	**0.946**	**0.966**	0.003
Yeo	**0.815**	**0.820**	**0.815**	**0.820**	**0.815**	**0.820**	**0.815**	**0.820**	0.002
Kumar	**0.989**	**0.993**	**0.989**	**0.993**	**0.989**	**0.993**	**0.989**	**0.993**	0.001
Buettner	0.442	0.452	0.498	0.550	0.479	0.515	**0.583**	**0.615**	0.000
mean	0.719	0.591	0.734	0.611	0.724	0.599	**0.745**	**0.627**	–

Table S10. Average clustering performance comparison of ranking-based feature selection methods on all datasets with different feature set sizes (20∼4000) in terms of ARI.

# genes	CellBRF	FEAST	HVGvst	HVGdisp	HRG
20	**0.5287±0.2545**	0.4301±0.2137	0.3257±0.1871	0.3136±0.2463	0.4152±0.2208
30	**0.5574±0.2486**	0.4609±0.2023	0.3561±0.2173	0.3472±0.2374	0.4537±0.2363
50	**0.6001±0.2262**	0.4887±0.1974	0.4157±0.2332	0.4037±0.2376	0.4564±0.2189
100	**0.5925±0.2108**	0.5286±0.1789	0.4611±0.2330	0.4690±0.2239	0.4872±0.1870
200	**0.6110±0.1872**	0.5421±0.1864	0.5186±0.2218	0.5032±0.2301	0.5272±0.1768
400	**0.5989±0.1866**	0.5486±0.1904	0.5413±0.2016	0.5440±0.1980	0.5233±0.1789
700	**0.5710±0.179**	0.5321±0.1850	0.5556±0.1963	0.5548±0.1854	0.5140±0.1882
1200	**0.5536±0.1822**	0.5365±0.1836	0.5524±0.2075	0.5509±0.1823	0.5144±0.1917
2000	0.5412±0.1851	0.5379±0.1859	0.5400±0.2066	**0.5514±0.1828**	0.5040±0.2016
4000	**0.5476±0.1824**	0.5311±0.1983	0.5252±0.2081	0.5295±0.1779	0.5083±0.1947

Table S11. Clustering performance comparison of feature sets with different feature set sizes (20∼4000 and three-sigma rule of thumb) on all datasets in terms of ARI.

Dateset	20	30	50	100	200	400	700	1200	2000	4000	3σ_rule
Buettner	0.189	0.252	0.169	0.353	0.624	**0.682**	0.635	0.66	0.642	0.68	0.615
Chu_celltime	0.689	0.687	0.694	0.723	0.724	0.711	0.716	0.734	0.732	0.721	**0.743**
Chung	0.18	0.197	**0.23**	0.136	0.157	0.14	0.145	0.133	0.124	0.151	0.172
Darmanis	0.552	0.638	**0.643**	0.633	0.594	0.618	0.587	0.594	0.587	0.583	0.625
Deng	0.399	0.479	0.439	0.441	0.457	0.46	0.445	0.447	**0.484**	0.465	0.449
Engel	0.698	0.731	0.726	0.766	0.759	0.696	0.715	0.678	0.71	0.662	**0.778**
Goolam	0.272	0.367	0.375	0.375	**0.468**	**0.468**	**0.468**	**0.468**	**0.468**	**0.468**	**0.468**
Kim	0.457	0.476	**0.541**	0.497	0.518	0.373	0.373	0.361	0.361	0.361	0.357
Koh	0.916	0.987	0.982	0.985	0.99	0.986	0.991	0.986	0.987	0.987	**0.991**
Kolodz	0.488	**0.534**	0.518	0.439	0.448	0.433	0.44	0.442	0.442	0.44	0.443
Kumar	0.986	0.979	0.978	0.978	0.978	0.978	**0.993**	**0.993**	0.978	0.971	**0.993**
Leng	0.944	0.966	0.955	**0.977**	0.932	0.911	0.755	0.78	0.681	0.651	0.966
Li	0.658	0.644	0.677	**0.721**	0.689	0.681	0.69	0.686	0.681	0.679	0.703
Maria2	0.411	0.385	0.392	0.427	**0.479**	0.423	0.439	0.471	0.439	0.433	0.438
Pollen	0.706	0.714	0.749	0.755	0.819	0.755	0.755	0.755	0.755	0.755	0.863
Robert	0.939	0.878	**0.959**	0.468	0.464	0.464	0.613	0.616	0.606	0.606	0.899
Ting	0.434	0.394	0.443	0.454	0.458	0.456	0.441	0.418	0.405	0.387	**0.462**
Treutlein	0.849	**0.87**	0.838	0.842	0.816	0.816	0.611	0.564	0.599	0.535	0.816
Usoskin	0.534	0.623	0.612	**0.73**	0.603	0.608	0.591	0.515	0.466	0.576	0.637
Yan	0.548	0.526	0.523	0.528	0.641	**0.691**	0.593	0.59	0.489	0.594	0.609
Yeo	0.783	0.819	**0.832**	0.728	0.728	0.642	0.602	0.602	0.707	0.717	0.82
Zhou	0.46	0.474	0.582	0.567	0.591	0.597	**0.613**	0.528	0.515	0.599	0.595
10X_PBMC	0.504	0.745	**0.753**	0.743	0.602	0.713	0.592	0.627	0.586	0.63	0.606
Cao	0.242	0.257	0.297	0.37	0.443	**0.448**	0.432	0.396	0.364	0.368	0.402
Chu_celltype	0.969	0.995	**0.996**	**0.996**	0.952	0.885	0.858	0.766	0.759	0.747	0.939
Han	0.339	0.439	**0.631**	0.579	0.617	0.572	0.622	0.54	0.519	0.538	0.594
Macosko	0.28	0.294	0.43	0.509	**0.542**	0.532	0.536	0.445	0.402	0.405	0.537
MacParland	0.254	0.312	0.387	0.458	0.418	0.514	0.421	0.397	0.368	0.347	**0.524**
Maria1	0.44	0.431	0.519	0.509	0.521	0.529	0.517	0.503	0.467	0.453	**0.533**
Puram	0.249	0.272	0.398	0.427	**0.491**	0.408	0.35	0.29	0.284	0.278	0.479
Shekhar	0.15	0.168	0.432	0.565	0.666	0.642	0.458	0.426	0.426	0.395	**0.675**
Yang	0.636	0.507	**0.644**	0.445	0.427	0.437	0.403	0.405	0.425	0.459	0.411
Zelsel	0.292	0.352	0.46	0.427	**0.547**	0.496	0.445	0.454	0.403	0.428	0.544

Table S12. Comparison of t-SNE embedding results based on silhouette coefficient and classifier accuracy on the time-course dataset.

	k					Mean	silhouette
	1	3	5	10	15		
CellBRF	**0.9404**	**0.9470**	**0.9536**	**0.9603**	**0.9603**	**0.9523**	**0.5557**
DUBStepR	0.9205	0.9007	0.9139	0.9073	0.9007	0.9086	0.4049
Feats	0.8609	0.8675	0.8940	0.9007	0.9073	0.8861	0.4645
FEAST	0.9205	0.9205	0.9073	0.9073	0.9007	0.9113	0.4444
geneBasisR	0.8675	0.8808	0.9139	0.8940	0.8874	0.8887	0.4334
HVG	0.8940	0.9007	0.8940	0.8874	0.8675	0.8887	0.3293
HRG	0.9139	0.9007	0.9139	0.9139	0.9205	0.9126	0.4480

Table S13. Comparison of t-SNE embedding results based on silhouette coefficient and classifier accuracy on the human tumor dataset.

	k					Mean	silhouette
	1	3	5	10	15		
CellBRF	**0.9881**	**0.9926**	**0.9926**	0.9881	0.9881	**0.9899**	**0.4354**
DUBStepR	0.9851	0.9866	0.9881	0.9881	0.9881	0.9872	0.3397
Feats	0.9673	0.9777	0.9821	0.9807	0.9792	0.9774	0.3255
FEAST	0.9792	0.9866	0.9881	0.9821	0.9807	0.9833	0.3730
geneBasisR	0.9628	0.9688	0.9702	0.9732	0.9702	0.9690	0.2074
HVG	0.9792	**0.9926**	**0.9926**	**0.9926**	**0.9926**	**0.9899**	0.3776
HRG	0.9836	0.9881	0.9881	0.9851	0.9836	0.9857	0.3267

Table S14. The runtime (in seconds) of various feature selection methods on five datasets of different sizes.

	#cells	CellBRF	DUBStepR	Feats	FEAST	geneBasisR	HVG	HRG
Chung	515	2.98	646.46	64.69	3.21	159.54	1.36	13.06
Chu2	1018	3.70	971.17	113.66	8.52	230.14	2.64	21.98
Han	2746	5.79	112.17	86.21	49.26	179.39	2.13	54.23
Macosko	14653	35.29	459.94	4643.04	72.56	1037.58	6.96	116.96
Shekhar	27499	73.32	310.20	7190.00	107.74	1581.83	9.08	343.41

Section A: Stability of CellBRF with respect to parameters

To evaluate the stability of CellBRF in terms of parameters, we divide the main parameters into four groups in Table S15 according to the steps in CellBRF. Then, we use different values to test each parameter while keeping the values of all other parameters unchanged on five gold-standard scRNA-seq datasets (Supplementary Fig. S4).

Table S15. A summary list of main parameters used in CellBRF.

Groups	Parameters	Brief introduction	Value
Group 1: spectral clustering-based cell cluster label prediction	P	the number of principal components (PC) to use	50
	k	the number of nearest neighbors used for k-NN graph construction	15
	n	the number of clusters	the number of types
Group 2: data balancing	h	the threshold to determine if a cluster is rare, central or major	$\frac{c}{n}$
	U	the fraction retained in major clusters	80%
	R	the sampling rate in SMOTE	$\frac{T_{C_{\text{central}}}}{T_{C_{\text{rare}}}}$
Group 3: random forest-based gene importance assessment	$n\_estimators$	the number of trees in the forest	1000
	bootstrap	whether bootstrap samples are used when building trees	FALSE
	...	additional parameters in the random forest model	defalut
Group 4: redundant gene removal based on linear similarity	h'	the threshold to remove highly redundant genes	$v + \frac{v'}{L+1}$

Group 1: spectral clustering-based cell cluster label prediction

There are three parameters in group 1, which are the number of principal components used in PCA (P), the number of neighbors (k) in k-NN, and the number of clusters used for clustering (n).

1. **the principal components (PCs) parameter** P: c) PCA is used to reduce the dimensionality of scRNA-seq data, containing all expressed genes, before constructing a k-NN graph. This overcomes the curse of dimensionality and enhances the efficiency of graph construction. The number of principal components used for analysis depends on the specific dataset and the

analysis goals, but it's common to use around 50 principal components in popular analysis tools like Scanpy and Seurat (Wolf F et al., 2018; Satija et al., 2015). So, we set P as 50 in CellBRF, consistent with prior studies. To investigate the impact of P, we run our model with the parameters 40, 45, 50, 55, and 60. Fig. S4a shows the clustering performance of the final selected genes with different parameter values in terms of NMI and ARI.

2. **the neighbor parameter** k: k is the number of neighbors in the k-NN algorithm for constructing the cell graph. It determines the number of edges adjacent to each node. In general, a larger k value leads to a more robust performance against noise but makes boundaries between clusters less distinct. For better clustering performance and consistency with previous studies (Wang et al., 2021; Yu et al., 2022), we set k to 15 in CellBRF to ensure a relatively complete network structure. To investigate the impact of k, we run our model with the parameters 5, 10, 15, 20, and 25. Fig. S4b shows the clustering performance of the final selected genes with different parameter values in terms of NMI and ARI. For better clustering performance and consistency with previous studies (Wang et al., 2021; Yu et al., 2022), we set k to 15 in CellBRF to ensure a relatively complete single-cell network structure.
3. **the number of clusters** n: n is the number of clusters used in spectral clustering, which determines the granularity or level of details at which the cells are segmented. The value of n in CellBRF is determined by the authors who generated the dataset. If this parameter is not provided, CellBRF can automatically determine an optimal number of clusters using the gap statistic approach. We consider five n values centered on n', that is, $n \in \{n'-2, n'-1, n', n'+1, n'+2\}$. Fig. S4c shows the clustering performance of the final selected genes with different parameter values in terms of NMI and ARI.

We use this spectral clustering flow to quickly obtain cell label assignment results with high accuracy. Therefore, although it may be better to use some data-driven strategies to guide the optimal selection of each data, a small amount of improvement consumes more computing time is unnecessary. Since these parameters primarily affect the accuracy of estimated cell-type labels, we randomly change the cell types of 0%, 5%, 10%, 15%, 20%, 50%, and 100% of cells and compare the clustering performance of the final selected genes in terms of NMI and ARI (Supplementary Fig. S5). Our experiments show that our method can select 50% of the reference genes and thus maintain stable clustering performance, provided that the cluster labels have some discriminative ability (NMI>0.2, ARI>0.2).

Group 2: data balancing

There are three parameters in group 2, which are the threshold to determine if a cluster is rare, central or major (h), the fraction retained in major clusters (U), and the sampling rate in SMOTE (R). Where the parameters h and R are automatically determined based on data. The border cells of the cluster

can be obtained through the cluster center, and removing these cells will not significantly affect the label accuracy, while retaining the specific information of the cluster, which is helpful for the feature selection step. To investigate the impact of U, we run CellBRF with varying values of U (70%, 75%, 80%, 85%, and 90%). Fig. S4d shows the NMI and ARI values obtained with different values of U. The best value is found at $U = 80\%$. Therefore, we set U as 80% in CellBRF.

Group 3: random forest-based gene importance assessment

The random forest model contains multiple parameters such as *n_estimators*, *bootstrap*, and so on. *n_estimators* is the number of trees in the forest. Using more trees in a random forest model can indeed make the model more stable and perform better in terms of accuracy and reducing overfitting. However, it's important to note that increasing the number of trees can also increase the computational time and memory required to build and use the model, which can slow down the model. After carefully considering the tradeoff between model performance and computational cost, we select 1000 trees in the random forest model. The parameter *bootstrap* is set to False, which means that we use the entire dataset to build each decision tree to ensure that the labels used for each tree are consistent in accuracy. For other parameters we use default values.

Group 4: redundant gene removal based on linear similarity

This group contains a dynamic threshold h' that gradually increases as more genes are removed. In this way, genes with high ranking and high correlation are filtered out. In order to investigate the impact of h', we run our model with the different initial correlation threshold v 0.7, 0.75, 0.8, 0.85, and 0.9. Fig. S4e shows the clustering performance of the final selected genes with different parameter values in terms of NMI and ARI. In contrast, it has better performance at 0.8. Therefore, we set the initial correlation threshold v as 0.8 in CellBRF.
